# The protective effect and antitumor activity of *Aconiti Lateralis Radix Praeparata* (Fuzi) polysaccharide on cyclophosphamide-induced immunosuppression in H22 tumor-bearing mice

**DOI:** 10.3389/fphar.2023.1151092

**Published:** 2023-03-24

**Authors:** Qi Hu, Yu Liu, Ji Yu, Xin Yang, Ming Yang, Yanan He, Li Han, Dingkun Zhang

**Affiliations:** ^1^ State Key Laboratory of Southwestern Chinese Medicine Resources, Pharmacy School, Chengdu University of Traditional Chinese Medicine, Chengdu, China; ^2^ State Key Laboratory of Innovation Medicine and High Efficiency and Energy Saving Pharmaceutical Equipment, Jiangxi University of Traditional Chinese Medicine, Nanchang, China

**Keywords:** Fuzi polysaccharide, immunomodulatory effect, structural characterization, liver cancer, combination medications

## Abstract

**Background:** *Aconiti Lateralis Radix Praeparata*, also known as Fuzi in Chinese, has been used in Traditional Chinese Medicine for more than 2,000 years. In recent years, some traditional herbal compounds containing Fuzi have achieved positive clinical results in tumor treatment. And the polysaccharide isolated from Fuzi has attracted much attention as a potential immunomodulator. However, its immunomodulatory mechanism remains to be further studied. Aim of the study. Fuzi neutral polysaccharide (FNPS) and cyclophosphamide (CTX) were combined to treat Hepatoma 22 (H22) tumor-bearing mice, and its mechanism of ameliorating immunosuppression caused by CTX was studied.

**Methods:** FNPS was isolated and purified. The molecular weight, functional groups, monosaccharide composition, and apparent morphology were characterized by gel permeation chromatography, Fourier transform infrared spectrometer, ion chromatography and scanning electron microscope, respectively. Through the analysis of tumor, immune organs, and serum cytokine levels of H22 tumor-bearing mice, the immunomodulatory effect and the protective effect on immunosuppressive mice induced by CTX was evaluated. And the immunomodulatory activity of FNPS was further verified by macrophage functional experiments.

**Results:** FNPS was composed of rhamnose, arabinose, galactose, glucose, and mannose in a molar ratio of 0.008:0.017:0.018:0.908:0.048. Its molecular weight was 94 kDa. *In vivo* experiments showed that 200 mg mL^−1^ FNPS could alleviate the suppression of immune organs and immune cells caused by CTX treatment, enhance the antitumor effect of CTX, increase the serum levels of Th1 immune-related pro-inflammatory cytokines (IL-1β and IL-6), and decrease Th2 immune-related anti-inflammatory cytokine (IL-10) and tumor-related pro-inflammatory cytokine (TNF-α) in the chemotherapy mice. Functional experiments revealed that 25 μg mL^−1^ FNPS could promote phagocytosis and proliferation of macrophages. When the concentration reached 50 μg mL^−1^, it enhanced the migration activity.

**Conclusion:** FNPS has the potential to alleviate the immunosuppressive effect of CTX by activating immune cells and promoting inflammation. It could be used as a potential auxiliary medication for liver cancer treatment.

## 1 Introduction

Liver cancer is the sixth most common fatal malignant tumor worldwide and the second leading cause of cancer death (Mohammadian et al., 2016). Due to the lack of specificity of liver cancer, patients are usually diagnosed at an advanced stage, which makes the window for surgical treatment lost (Xu et al., 2021). Chemotherapy and immunotherapy are the main options for liver cancer treatment at present. Cyclophosphamide (CTX) is recommended as the common drug in the treatment of liver cancer, including T-cell therapy (Shi et al., 2020), immunotherapy (Löffler et al., 2022), chemotherapy (Eklund et al., 2005) and liver transplantation (Ringdén et al., 2000). However, the immunosuppressive side effects caused by the CTX make its therapeutic prognosis poor, and prolonged suppression of the immune system can increase the risk of cancer development (Axelrad et al., 2016). It is necessary to mitigate the side effects of chemotherapy drugs.

Traditional Chinese Medicine has a long history of being used to treat cancer. Saponins, polysaccharides, polyphenols, and other active ingredients in Traditional Chinese Medicine have been reported to exert immunomodulatory activity (Shirani et al., 2015; He et al., 2022). It is an important way to improve the immunosuppression of chemotherapy drugs. Polysaccharides are the material basis for various herbs to exert their immunomodulatory activity (Zou et al., 2017; Liu et al., 2023). Pharmacological studies have proved that Astragalus polysaccharide could resist the immunosuppressive side effects of the chemotherapy drug Adriamycin (Li et al., 2008). The combination of fucoidan and chemotherapy drugs could inhibit tumor invasion and improve the survival rate of lung cancer patients (Hsu and Hwang, 2019). Sepia ink polysaccharides have the potential to increase the anti-tumor activity of CTX and reduce the thymus toxicity of CTX in mice (Li et al., 2018). Therefore, polysaccharides may be an effective component to resist the side effects of chemotherapy drugs.

As a valuable and important Traditional Chinese Medicine, *Aconiti Lateralis Radix Praeparata* (Fuzi) is often used for the treatment of heart failure, rheumatism, joint pain, gastroenteritis, and tumors (Zhang et al., 2016a; Zhang et al., 2016b; Qi et al., 2018; He et al., 2021). In the treatment of malignant tumors, Fuzi is believed to improve the prognosis of advanced primary liver cancer. After drug intervention, the median survival time of patients was extended from 4.85 to 6.74 months, further prolonging the life of patients (Liang and Hu, 2020). At the same time, Jiedu granules which contain Fuzi, had also been reported to prolong the median survival time of advanced liver cancer patients from 4.5 to 10.5 months (Yifu et al., 2022). Pharmacological studies proved that Fuzi exerts anti-tumor effects mainly by inhibiting the proliferation of tumor cells (Li, 2022), inducing the apoptosis of liver cancer cells (Liang et al., 2015), and activating reactive oxygen species (Qi et al., 2018). Polysaccharides in Fuzi was considered to inhibit the growth of H22 tumors in mice by improving peripheral blood leukocytes, promoting serum cytokine secretion, and improving the immune response (Li et al., 2013). In order to explore whether the combination of Fuzi neutral polysaccharide (FNPS) and CTX has the potential to relieve or alleviate the immunosuppressive side effects and enhance the efficacy, a neutral polysaccharide fragment from Fuzi was isolated. And preliminarily characterized it by gel permeation chromatography (GPC), Fourier transform infrared spectrometer (FT-IR), ion chromatography (IC) and scanning electron microscope (SEM). The immunomodulatory activity of FNPS was studied in RAW264.7 cells. In addition, the effects of the combination of FNPS and CTX were studied in H22 tumor-bearing mice. It provided scientific support for the development and application of adjuvant drugs in the treatment of liver cancer.

## 2 Materials and methods

### 2.1 Materials

#### 2.1.1 Drug and reagents

Raw slices of Fuzi were purchased from Sichuan Good doctor Panxi Pharmaceutical Co., Ltd. (Xichang, China). DEAE-52 cellulose was provided by BoRui Saccharide Biotech Co., Ltd. (Yangzhou, China). G-100 Sephadex was obtained from Shanghai Yuanye Bio-Technology Co., Ltd. (Shanghai, China). CTX was gotten from Shanghai Yien Chemical Technology Co., Ltd. (Shanghai, China). RPMI 1640 medium was purchased from Thermo Fisher Scientific (Shanghai, China). Fetal bovine serum (FBS) was provided by Zhejiang Tianhang Biotechnology Co., Ltd. (Huzhou, China). IL-1β, IL-6, IL-10, and TNF-α cytokine detection kits were obtained from MultiSciences (Lianke) Biotech Co., Ltd. (Hangzhou, China). DEME high glucose medium, Lipopolysaccharide (LPS), phosphate buffer solution (PBS), and lactate dehydrogenase (LDH) detection kit were purchased from Wuhan Servicebio Technology Co., Ltd. (Wuhan, China). Biosharp Cell Counting Kit- 8 (CCK-8) and red were purchased from Beijing Labgic Technology Co., Ltd. (Beijing, China). All other reagents were analytical grade.

#### 2.1.2 Animals

Male ICR mice (6–8 weeks old) weighing 20 ± 2 g were purchased from SPF (Beijing) Biotechnology Co., Ltd. [Certificate No. SCXK (Jing) 2019–0010, Beijing, China].

### 2.2 Ethics approval

The study was conducted strictly in accordance with the recommendations of the Guidelines for the Care and Use of Laboratory Animals issued by the Ministry of Science and Technology of China. This experiment was approved by the Ethical Committee of Affiliated Hospital of Chengdu University of Traditional Chinese Medicine (Approval ID: 2017BL-003).

### 2.3 Study on the structure of FNPS

#### 2.3.1 Extraction and purification of FNPS

**FIGURE 1:**
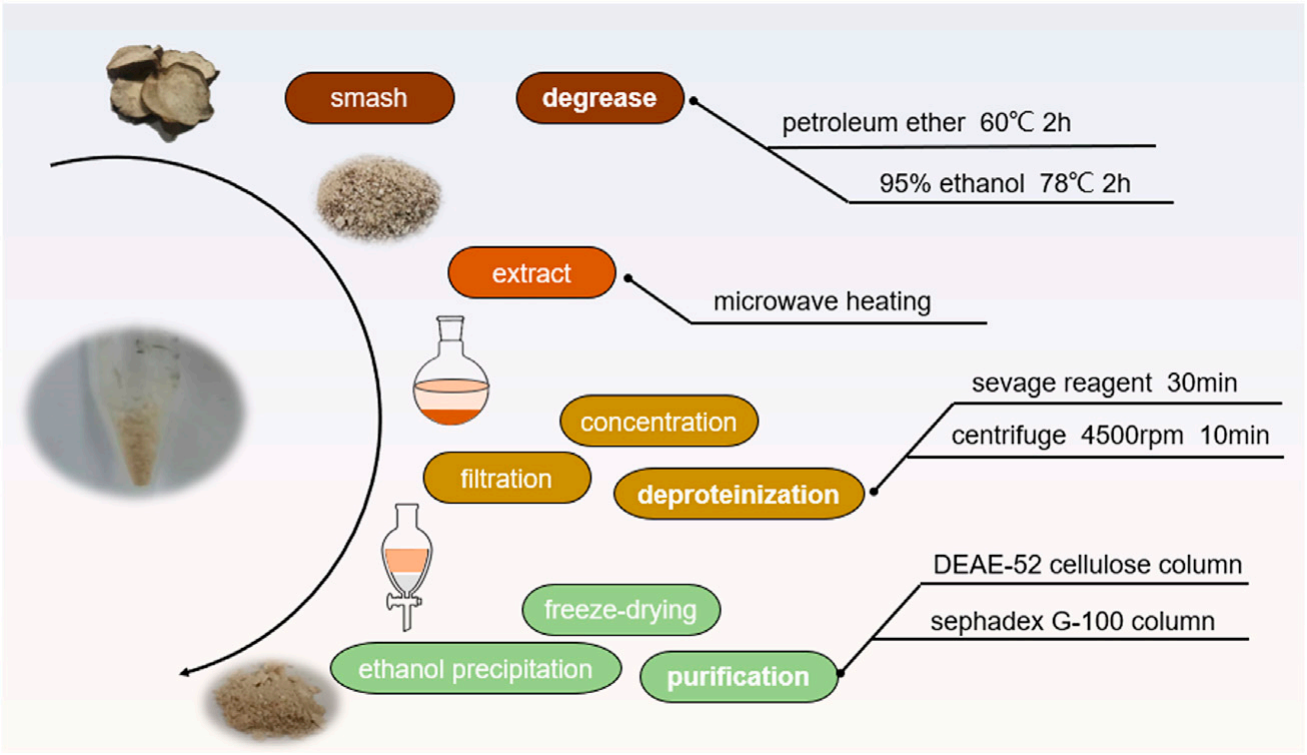
Extraction process of FNPS.

The extraction process of FNPS was shown in Figure 1. It was performed based on the reported method with minor modifications (Yang et al., 2020). Raw slices of Fuzi were powdered, added ten times the amount of petroleum ether, refluxed three times in a 60°C water bath for 2 h, and discarded the extraction. Then added with ethanol, and refluxed twice in a 78°C water bath for 2 h. Evaporate the solvent to obtain skimmed Fuzi. Dry skimmed Fuzi was soaked in pure water for microwave extraction of polysaccharides. The extract was dried and redissolved in water, then deproteinized by 1/5 volume of sevag reagent (trichloromethane: n-butanol = 5:1, v/v). Concentrated under reduced pressure, and added with ethanol into the extract to reach a final concentration of 80% ethanol, which was placed in the refrigerator at 4°C overnight. The precipitation was collected and redissolved in water, and freeze-dried to obtain FNPS. Subsequently, it was separated and purified by DEAE-52 cellulose which was eluted by 0, 0.1, 0.2 M NaCl gradient, and G-100 Sephadex column which was eluted by pure water. The phenol-sulfuric acid method was used to monitor the purification process. The absorbance was measured at a wavelength of 490 nm, and the elution curve was drawn. Purified FNPS was obtained after freeze-drying. The purity of FNPS was expressed by total sugar content.

#### 2.3.2 Chemical composition analysis

The content of total sugar in purified FNPS was determined by the phenol-sulfuric acid method (using glucose as standard). The Coomassie Brilliant Blue method (using bovine serum albumin as standard) was used to assess protein content. Glucuronic acid content was determined using the m-hydroxydiphenyl method (using glucuronic acid as the standard) (Bai et al., 2022). Full-wavelength scan of UV-visible spectrophotometry was recorded (A560, Aoyi Instrument Shanghai Co., Ltd.), and nucleic acids and proteins of FNPS were determined at wavelengths of 260 and 280 nm.

#### 2.3.3 Infrared spectroscopy

FT-IR spectra were collected over a wavelength range of 4000–400 cm^−1^ (Nicolet 670, Nicolet, United States). 1 mg FNPS powder and 200 mg KBr were finely ground and pressed into a transparent thin slice. Put it into the infrared spectrometer for testing. The wavenumber range was set at 4000–400 cm^−1^, the number of scans was 32, and the resolution was 4 cm^−1^.

#### 2.3.4 Molecular weight determination

The molecular weight of FNPS was determined by GPC. FNPS was prepared as a 1.59 mg mL^-1^ solution and applied to an Agilent PL-GPC50/Agilent 1260 system equipped with a PL aquagel-OH MIXED column (8 μm, 7.5 × 300 mm) and a light scattering detector. The mobile phase was 0.15 M NaNO_3_, the injection volume was 50 μL, the flow rate was 1 mL min^-1^, and the column temperature was 30°C.

#### 2.3.5 Monosaccharide composition

The monosaccharide composition of FNPS was detected using IC (ThermoFisher ICS5000, United States). The mixed standard stock solution was prepared from 16 monosaccharide standards (fucose, rhamnose, arabinose, galactose, glucose, xylose, mannose, fructose, ribose, galacturonic acid, glucuronic acid, galactosamine hydrochloride, glucosamine hydrochloride, N-acetyl-D glucosamine, guluronic acid, mannuronic acid). FNPS (5 mg) was hydrolyzed by heating with 2 mL 3 M trifluoroacetic acid for 3 h at 110°C. The solution was blow-dried with nitrogen, and 5 mL water was added. Mixed well with vortex. Pipetting 50 µL solution and 950 µL deionized water into a centrifuge tube. Supernatants were collected by centrifugation (12,000 rpm, 5 min) and injected for IC analysis.

#### 2.3.6 Scanning electron microscopy

Before SEM observation, a layer of platinum was sprayed on the surface of the sample. The structure of the sample was analyzed by SEM (ZEISS MERLIN Compact, Zeiss, Germany) at a voltage of 10 kV and the surface morphology of purified FNPS was observed at different magnifications.

#### 2.3.7 NMR spectroscopy

50 mg FNPS were dissolved in D_2_O for testing. The 1D (^1^H, ^13^C) and 2D (HSQC, ^1^H-^1^H COSY) NMR spectra of FNPS were collected by a NMR sprctrometer (Bruker avance neo 400M, Bruck, Germany).

### 2.4 Effect of FNPS combined with cyclophosphamide on H22 tumor-bearing mice

#### 2.4.1 Establishment of tumor model

To verify whether FNPS could enhance the anti-tumor effect and reduce the immunosuppressive effect of CTX, an H22 tumor-bearing mice model was established. All male ICR mice were divided into 5 groups (control group, model group, CTX group, low-dose combination group, and high-dose combination group) randomly (*n* = 8 per group). H22 liver cancer cells (1 × 10^6^ cells per mouse) in the logarithmic growth phase were inoculated subcutaneously to ICR mice at the right axilla. The administration was started after 3 days of adaptive feeding in mice.

#### 2.4.2 Administration

The control group and the model group were given normal saline. The CTX group was given 30 mg·(kg·d)^−1^ CTX, oral administration for 7 days. The low-dose combination group was given 30 mg·(kg·d)^−1^ CTX and 100 mg·(kg·d)^−1^ FNPS. The high-dose combination group was given 30 mg·(kg·d)^−1^ CTX and 200 mg·(kg·d)^−1^ FNPS. Administered orally for 7 days with free access to water during the experiment.

#### 2.4.3 Determination of tumor inhibition rate and immune organ index

After the last day of administration, the mice were weighed on the digital weighing scale. The blood was taken from the eyeballs and then sacrificed by cervical dislocation. After dissection, the thymus, spleen, and tumor tissues were collected. Washed with sterile saline, drained with filter paper, weighed, and recorded. Tumor inhibition rate and immune organ index were calculated as follows (Liu et al., 2022):

$$Tumor\;inhibition\;rate\;\left(\%\right)=\frac{W\;\left(model\;group\right)-W\;\left(experimental\;group\right)}{W\;\left(model\;group\right)}\times 100\%$$

$$Thymus\;index=\frac{W\;\left(thymus\;weight\right)}{W\;\left(mice\;weight\right)}$$

$$Spleen\;index=\frac{W\;\left(spleen\;weight\right)}{W\;\left(mice\;weight\right)}$$

#### 2.4.4 Lymphoproliferation

According to the method reported in the literature (Yu et al., 2018), but slightly modified, the proliferation activity of spleen lymphocytes was determined by the CCK-8 method. After the mice were sacrificed, the spleen tissues were extracted under aseptic conditions. Placing spleen in culture dishes containing red blood cell lysate buffer and PBS, and transferring to RPMI-1640 medium containing 10% FBS and 1% penicillin/streptomycin. After 48 h of culture, cells were plated in a 96-well plate. Lymphocytes were stimulated with LPS. CCK-8 was added for 4 h, and absorbance was measured at 450 nm.

$$Lymphocyte\;proliferation\;rate=\frac{OD\;\left(LPS\;stimulated\right)-OD\left(no\;cell\;treatment\right)}{OD\;\left(no\;LPS\;stimulated\right)-OD\left(no\;cell\;treatment\right)}$$

#### 2.4.5 H-E staining of tumor tissue

After mice were sacrificed, tumor tissues were removed and fixed with paraformaldehyde. Paraffin sections were prepared from tumor tissues after dehydration and embedding. And the sections were deparaffinized in xylene, hydrated in graded alcohol solutions, and stained with hematoxylin and eosin staining solutions. The pathological changes of tumor tissues in each group were observed under a light microscope.

#### 2.4.6 Enzyme-linked immunosorbent assay

After blood collection from the eyeballs of mice, blood samples were placed at room temperature for 1 h and centrifuged at 3500 rpm for 10 min to separate serum. Levels of IL-1β, IL-6, IL-10, and TNF-α in serum were measured using designated Elisa kits. Cytokine levels were expressed as picograms per milliliter of serum according to an appropriate standard curve.

### 2.5 Immunomodulatory function of FNPS *in vitro*

#### 2.5.1 Cell culture

RAW264.7 cells were obtained from ATCC (American Type Culture Collection). They were cultured and subcultured in DEME high glucose medium added with 10% FBS and 1% penicillin/streptomycin. Cells were grown at 37°C with 5% CO_2_ in humidified air. 1 μg mL^−1^ LPS dissolved in a complete medium served as the positive control. 10 mg FNPS was dissolved in a 10 mL complete medium to prepare the stock solution.

#### 2.5.2 Cell viability

Cell viability was determined using the CCK-8 method. RAW264.7 cells were adjusted to a density of 5 × 10^4^ and seeded in a 96-well plate and incubated for 24 h to attach. Cells were treated with different concentrations of FNPS (25, 50, 100, 200, and 400 μg mL^-1^), a positive control (1 μg mL^−1^ LPS) and a sample control (no drug) were set. After 24 h, 10 μL CCK-8 was added and incubated for 2 h to measure the absorbance at 450 nm.

$$Cell\;viability\;\left(\%\right)=\frac{OD\;\left(experimental\;group\right)}{OD\;\left(sample\;control\;group\right)}\times 100\%$$

#### 2.5.3 Lactate dehydrogenase release

The LDH level in the cell culture supernatant was determined using an LDH assay kit. RAW264.7 cells were grown and treated in a 96-well plate. Blank control wells (no cells), sample control wells (no drug), sample maximum enzyme activity control wells, and experimental wells were set. After 24 h, Cell lysis buffer was added to maximum enzyme activity control wells. Centrifuge the supernatant for 5 min at 1,000 rpm. The chromogenic agent from the LDH assay kit was added and incubated for 30 min in an incubator to determine the absorbance at 490 nm.

$$Cytotoxicity\;\left(\%\right)=\frac{OD\;\left(experimental\;group\right)-OD\;\left(sample\;control\;group\right)}{OD\;\left(OD\;maximum\;enzyme\;activity\;group\right)-\left(sample\;control\;group\right)}\times 100\%$$

#### 2.5.4 Phagocytic function

The neutral red assay was used to determine the effect of FNPS on the phagocytic function of RAW264.7 cells. The sample control group (no drug), the positive control group (1 μg mL^-1^ LPS), and the experimental group (25, 50, and 100 μg mL^-1^ FNPS) were set. The medium was removed after 24 h incubation, and neutral red dye solution (100 μL·well^-1^) was added for 3 h, PBS washed and cell lysis buffer (absolute ethanol: acetic acid = 1:1) was added. Absorbance at 570 nm was measured after incubation at room temperature for 30 min. The phagocytic index was calculated using the following formula (Liu et al., 2021):

$$Phagocytic\;index=\frac{OD\;\left(experimental\;group\right)}{OD\;\left(sample\;control\;group\right)}$$

#### 2.5.5 Migration function

The migration ability of RAW264.7 cells was determined using a Transwell chamber (3 μm pores). RAW264.7 cells were seeded in a 12-well plate at a density of 5 × 10^4^/well and incubated for 24 h to attach. The cells were treated with different concentrations of FNPS (25, 50, and 100 μg mL^-1^), and 1 μg mL^-1^ LPS was set as a positive control. 200 μL cell suspension was added to the upper chamber, and 700 μL 20% FBS was added to the lower chamber. After being cultured in the cell incubator for 24 h, the non-migrated cells in the upper chamber were wiped off with a cotton swab. Fixed with paraformaldehyde, PBS washed and stained with crystal violet staining solution. Five areas were selected randomly to capture and analyzed with Image J software.

### 2.6 Statistical analysis

The data were presented as mean ± standard deviation (SD). All Statistical analysis was performed using IBM SPSS Statistics 27 (IBM SPSS Statistics, IBM Corporation, Armonk, NY, United States). The analysis of variance (ANOVA) followed by the LSD *post hoc* test was used for multiple-comparison between groups. *p* < 0.05 was considered a statistically significant difference when compared to the negative, positive, or model group.

## 3 Results

### 3.1 Characteristics of FNPS

**FIGURE 2:**
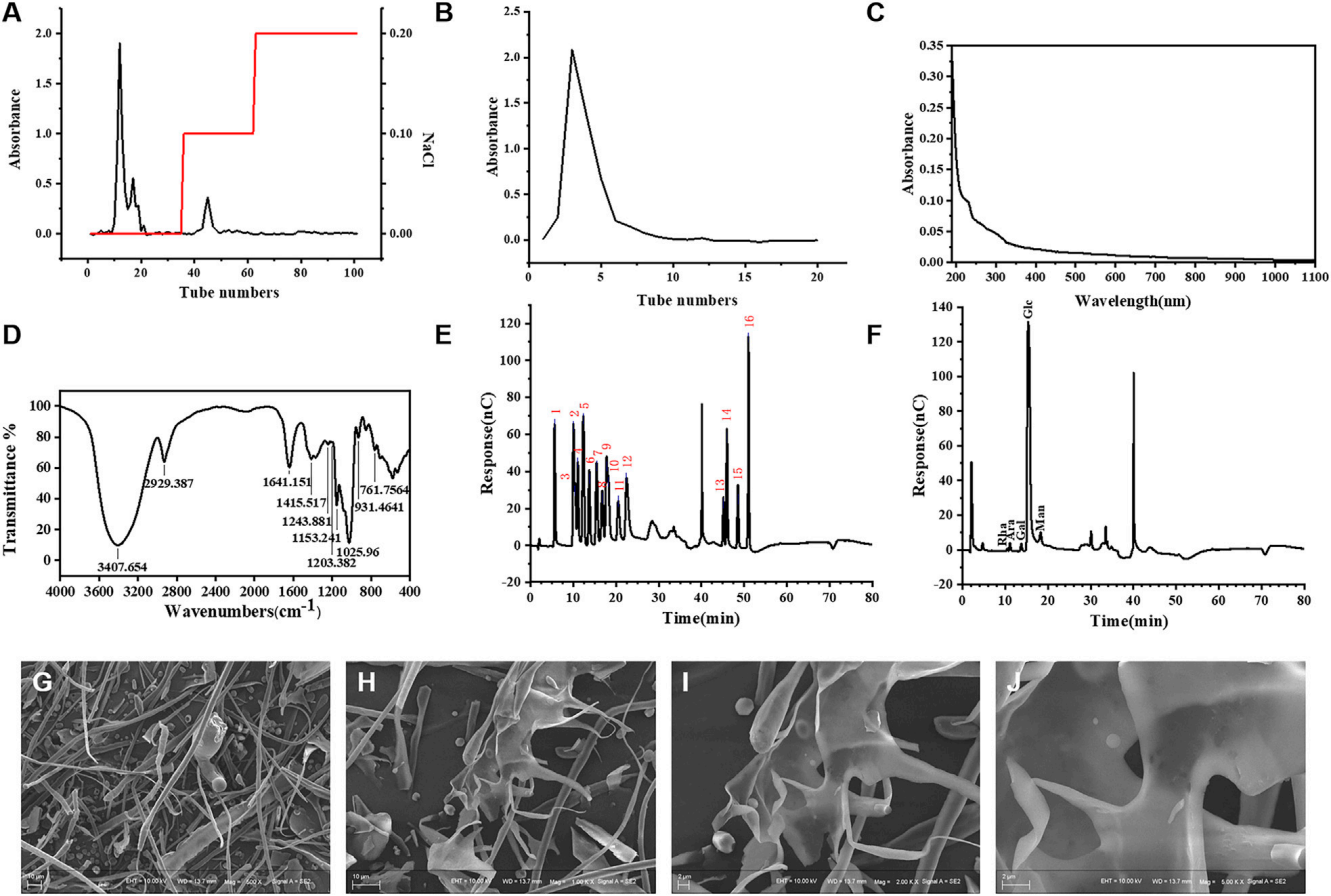
Structure characterization of FNPS. Elution profile of FNPS on DEAE-52 cellulose column **(A)** and Sephadex G-100 column **(B)**. **(C)** UV–vis spectrum of FNPS. **(D)** Infrared spectra analysis of FNPS. Ion chromatogram of sixteen mixed standards **(E)** and FNPS **(F)**. Magnification: **(G)** ×500; **(H)** ×1000; **(I)** ×2000; **(J)** ×5000.

FNPS was further purified by the DEAE-52 cellulose column. The elution curve was shown in Figure 2A. After gradient elution with purified water, 0.1 M, and 0.2 M NaCl solution, two main peaks were obtained. One was the peak of neutral polysaccharide eluted with deionized water and another one was the peak of acidic polysaccharide eluted with 0.1 M NaCl. The main component (neutral polysaccharide) was further purified by the Sephadex G-100 column. The elution curve is shown in Figure 2B. The peak components were concentrated and freeze-dried to obtain uniform polysaccharide fractions.

According to the determination method, the contents of total sugar and uronic acid of FNPS were 1.02 mg mg^-1^ and 0.0704 mg mg^-1^, and no protein component was detected. In addition, the UV-VIS spectra of FNPS did not show absorption peaks at 260 and 280 nm (Figure 2C). 280 nm is often used to indicate the absorbance of proteins and 260 nm for nucleic acids. The results showed that there was no nucleic acid and protein in the purified FNPS.

FT-IR spectra revealed the main functional groups of FNPS (Figure 2D). A wide and strong peak at 3407 and 2929 cm^−1^ was assigned to the stretching vibration of O-H and C-H (Ren and Liu, 2020). The stretching vibration at 1641 cm^−1^ was assigned to O-H (Song et al., 2020). The one at 1415 cm^−1^ was assigned to the stretching vibration of C-H or the bending vibration of O-H (Wu et al., 2019). The absorption peaks at 1200–1000 cm^-1^ could be assigned to the stretching vibration of C-O (Wang et al., 2020). And a peak at 931 cm^-1^ was owing to the α-glucoside bond (Mutaillifu et al., 2020). The above results indicated that the extracted component was polysaccharide. The Mp, Mw, and Mn were 41,739 Da, 94,564 Da, and 36,026 Da, respectively. The polydispersity was 2.625.

Figure 2E showed the IC chromatogram of the standard solution. 2.0 min was the peak of sodium hydroxide, and 41.0 min was the peak of sodium acetate. From left to right were fucose (5.569 min), galactosamine hydrochloride (10.084 min), rhamnose (10.5 min), arabinose (11.117 min), glucosamine hydrochloride (12.367 min), galactose (13.784 min), glucose (15.409 min), N- Acetyl-d glucosamine (16.792 min), xylose (17.834 min), mannose (18.284 min), fructose (20.534 min), ribose (22.484 min), galacturonic acid (45.125 min), glucuronic acid (45.95 min), glucuronic acid (48.509 min), and mannuronic acid (50.992 min). The IC chromatogram of FNPS (Figure 2F) showed absorption peaks at 10.5, 11.117, 13.784, 15.409, and 18.284 min, indicating that FNPS was composed of rhamnose, arabinose, galactose, glucose, and mannose. The mole ratio was 0.008:0.017:0.018:0.908:0.048.

The microstructure of FNPS was detected by SEM. At ×500 magnification, FNPS appeared as a filamentous structure (Figure 2G). After magnification to ×1000, it could be found that it was doped with fine particles and an irregular sheet structure (Figure 2H). Magnification to ×2000 revealed obvious folds on the surface of the irregular sheet structure, in which fine particles could be embedded (Figure 2I). After magnification to ×5000, FNPS displayed a sheet-like porous structure (Figure 2J).

**FIGURE 3:**
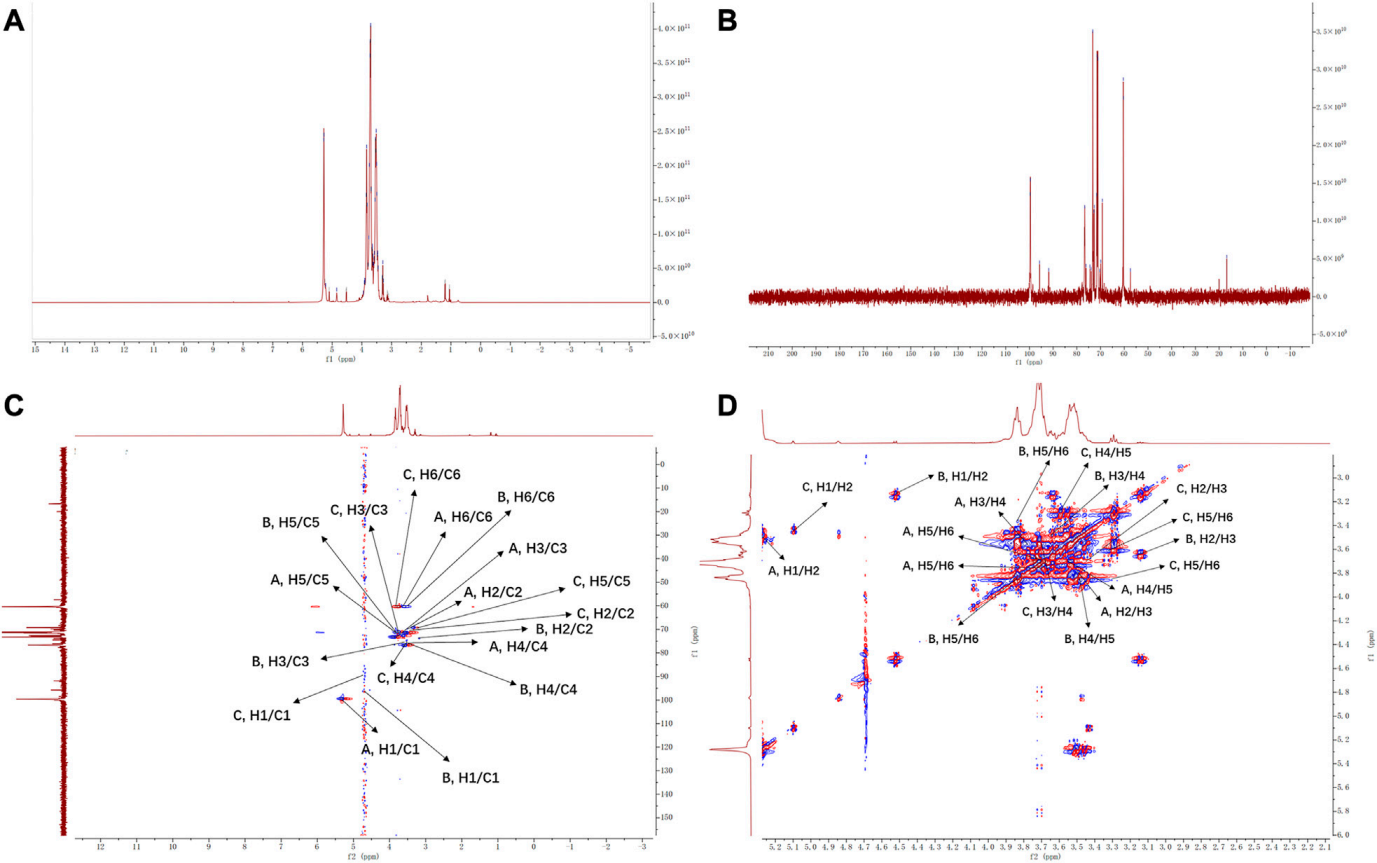
NMR spectrum of FNPS. **(A)** 1H NMR. **(B)** 13C NMR. **(C)** HSQC spectrum. **(D)** ^1^H-^1^H COSY spectrum.

According to 1D and 2D NMR data (Figure 3), there are three heterocephalic proton signals in δ 5.28, 5.09, 4.52 ppm, respectively. Suggesting that there are α and β glycosidic bond in FNPS. Three signal peaks were shown in the heteroperic carbon signal region of FNPS (δ 99.69, 95.72, 91.85 ppm, respectively). Combined with HSQC spectrum, three groups of different heterosignals can be distinguished, which are 5.28/99.91, 4.52/95.72, 5.09/91.85, respectively. They are named as residues A, B and C, and classified according to the cross-peaks shown in ^1^H-^1^H COSY spectrum. Based on the literature (Zhao et al., 2006; Yang et al., 2020), the chemical shift of sugar residues was attributed. The corresponding chemical shifts of residue are shown in Table 1. It is speculated that FNPS is a glucan, which is mainly linked by α-1, 4-glucoside bond.

**TABLE 1:** ^1^H and^13^C NMR chemical shifts of FNPS.

Residue	Δ^1^H/^13^C (ppm)						
	H1/C1	H2/C2	H3/H3	H4/C4	H5/C5	H6a/C6	H6b
A, →4) - Α - D -GLC -(1 →	5.28	3.50	3.83	3.55	3.71	3.68	3.76
	99.91	71.89	73.11	76.51	72.24	60.57	
B, →4) - Β - D -GLC	4.52	3.16	3.65	3.49	3.84	3.56	3.85
	95.72	73.90	76.43	76.69	71.37	60.35	
C, →4) -Α- D -GLC	5.09	3.45	3.70	3.58	3.29	3.85	3.60
	91.85	71.98	72.50	76.95	69.19	60.57	

### 3.2 Tumor inhibitory rate and immune organ index

**FIGURE 4:**
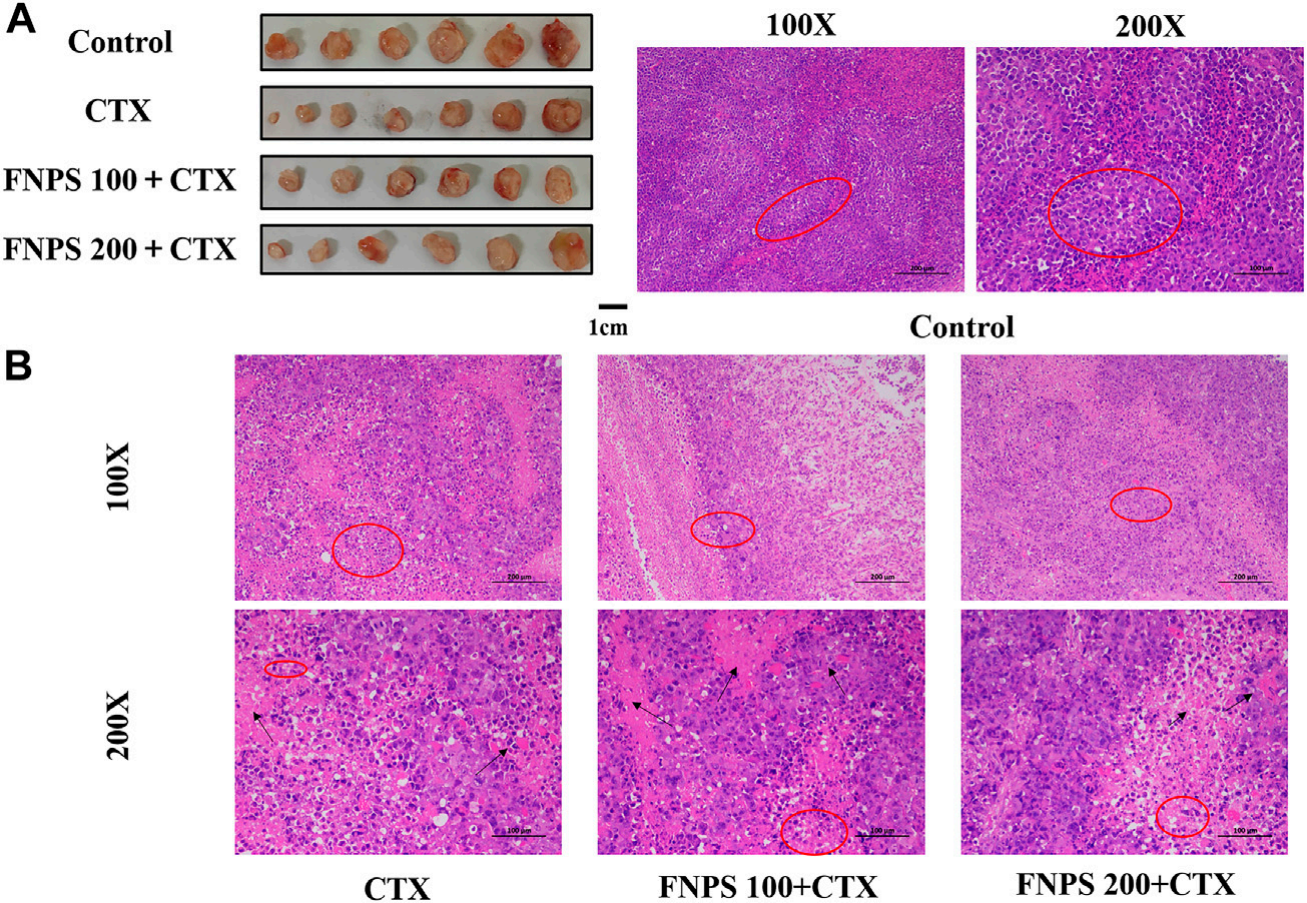
Synergistic anti-tumor effect of FNPS. **(A)** Representative photographs of the tumor of mice. **(B)** The images of H-E staining for tumor tissue. Note: Control, normal mice; Model, untreated tumor model mice; CTX, cyclophosphamide treated tumor model mice; *p < 0.05 *versus* Model, **p < 0.01 *versus* Model, ^#^ *p* < 0.05 *versus* CTX, ^##^ *p* < 0.01 *versus* CTX.

**FIGURE 5:**
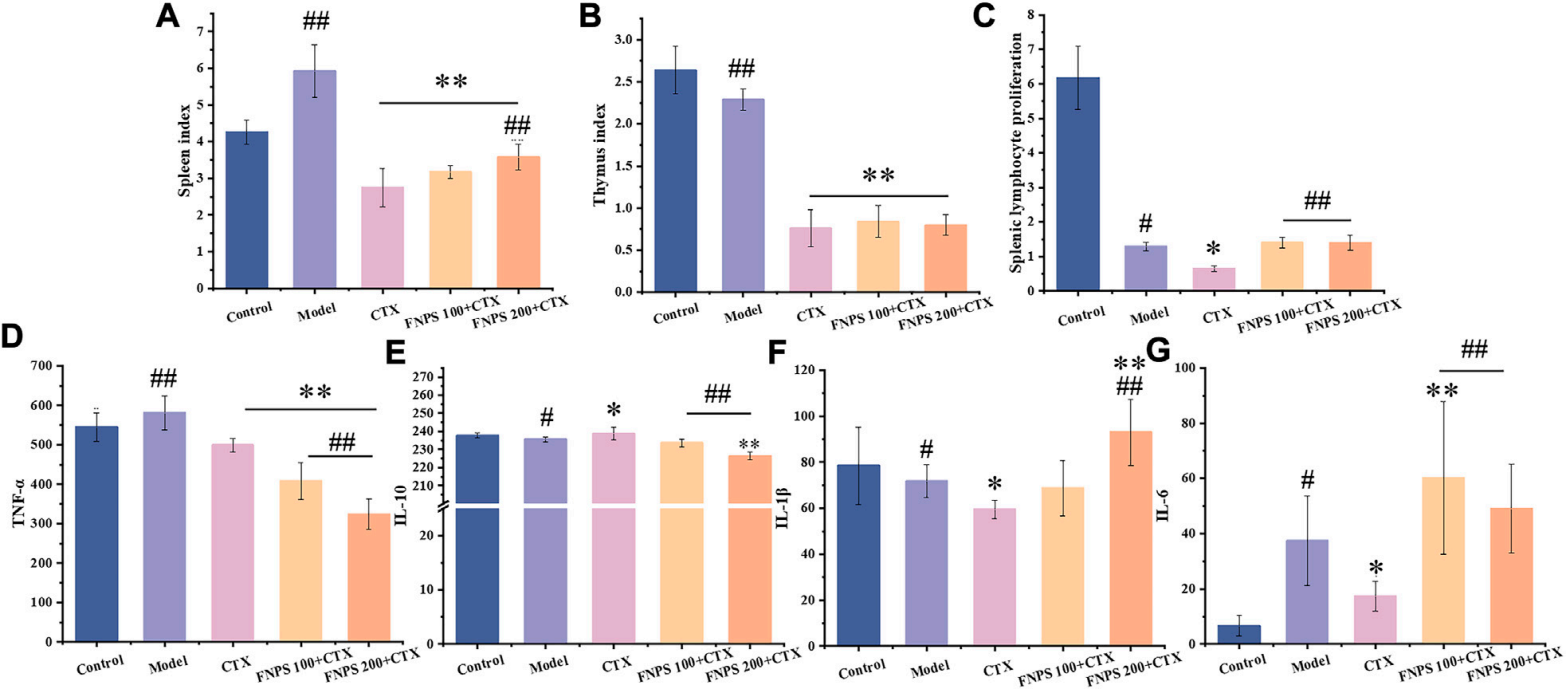
Effects of FNPS on H22 tumor-bearing mice treated with chemotherapy. **(A)** Effects of FNPS on spleen index. **(B)** Effects of FNPS on thymus index. **(C)** FNPS reduces the inhibitory effect of chemotherapy on lymphocyte proliferation. **(D)** TNF-α levels in the serum. **(E)** IL-10 levels in the serum. **(F)** IL-1β levels in the serum. **(G)** IL-6 levels in the serum. Note: Control, normal mice; Model, untreated tumor model mice; CTX, cyclophosphamide treated tumor model mice; *p < 0.05 *versus* Model, **p < 0.01 *versus* Model, ^#^ *p* < 0.05 *versus* CTX, ^##^ *p* < 0.01 *versus* CTX.

The tumor size of the H22 tumor-bearing mice was shown in Figure 4A. All drug treatment groups exerted inhibitory effects on the growth of H22 solid tumors in mice. After CTX treatment, the tumor inhibitory rate was 16.19%, the tumor inhibitory rate of the 100 mg·(kg·d)^−1^ FNPS group was increased to 21.53%, and the tumor inhibitory rate of the 200 mg·(kg·d)^−1^ FNPS group was increased to 25.84%. The thymus and spleen are important immune organs in the body. The weight of the thymus and spleen could reflect the immune status of the body. The spleen index and the thymus index were shown in Figures 5A, B. Compared with the control group, the tumor growth caused spleen enlargement and thymus atrophy, and the immune function of mice was disturbed. The indexes of the thymus and spleen in the CTX group were the lowest, which were significantly lower than those in the model group (*p* < 0.01). It indicated that CTX treatment had a strong toxic effect on immune organs, resulting in immunosuppression. The combined administration of 200 mg·(kg·d)^−1^ FNPS significantly alleviated the toxicity of CTX to immune organs (*p* < 0.05).

### 3.3 H-E staining of tumor tissue

The H-E staining results of tumor tissue were shown in Figure 4B. In the model group, the tumor cell had a high density, round shape, and deep nuclear staining. The boundary between tumor cells and stroma was not clear. The tumor cells in the CTX group and the FNPS combined administration group were scattered, with an irregular morphology, uneven size, and constricted nuclear. And there were necrotic areas in the tumor tissue. It indicated that 100 mg·(kg·d)^−1^ combined administration of FNPS promoted the apoptosis and tissue necrosis of tumors induced by CTX. H-E staining results further confirmed that FNPS could inhibit the growth of tumor cells.

### 3.4 Lymphocyte proliferation activity

The result of the lymphocyte proliferation test was shown in Figure 5C. The results indicated that the splenic lymphocyte proliferation capacity of the experimental group was significantly lower than that of the control group (*p* < 0.01). Lymphocyte proliferation was significantly reduced after CTX treatment compared with the model group (*p* < 0.05), which indicated that CTX has a non-selective inhibition on lymphocytes in the body. Compared with the CTX group, the combined administration of 100 mg·(kg·d)^−1^ FNPS could significantly enhance the proliferation ability of lymphocytes (*p* < 0.01), which illustrated that FNPS could activate splenic lymphocyte immune response and enhance cellular immunity.

### 3.5 Serum cytokine levels

Inflammatory factors are important cytokines involved in immune regulation, and the level can reflect the immune status of mice. TNF-α is an important factor involved in the pro-inflammatory response, which can promote tumor growth and poor prognosis of liver cancer (Jing et al., 2018). The levels of TNF-α, IL-10, IL-1β, and IL-6 in the serum of each group of mice were shown in Figures 5D–G. TNF-α levels were elevated in tumor mice compared to the control group. It was associated with tumor growth in mice. All treatment groups showed a tendency to lower TNF-α levels, which was due to the inhibition of tumor growth. A dose-dependent decrease in TNF-α was shown after FNPS administration (*p* < 0.01). This may be related to the inhibition of the tumor and the reduction of tumor inflammation. IL-10 is a cytokine with an anti-inflammatory property that limits the body’s immune response to pathogens (Saraiva and O'Garra, 2010). There was no significant change in IL-10 level after the tumor, but the level of IL-10 increased after CTX administration. It showed that CTX limited the immune response to the pathogen. After FNPS combined administration, IL-10 level significantly decreased in a dose-dependent manner (*p* < 0.01). IL-1β and IL-6 are cytokines associated with inflammation and are often used to evaluate the ability of drugs to stimulate an immune response (Ni et al., 2016). After CTX administration, IL-1β was significantly decreased (*p* < 0.05), which was associated with immunosuppression induced by CTX. IL-1β increased in a dose-dependent manner after FNPS combined administration. After 200 mg·(kg·d)^−1^ FNPS combined administration, the IL-1β level was significantly increased (*p* < 0.01). IL-6 levels increased significantly after the tumor (*p* < 0.05). It was associated with the development of liver cancer. After CTX administration, IL-6 levels were significantly decreased (*p* < 0.05), which was related to the therapeutic effect of CTX on tumors and immunosuppression side effects. After 100 mg·(kg·d)^−1^ FNPS combined administration, the IL-6 level increased (*p* < 0.01). This is related to the protective effect of polysaccharides on the immunosuppression of CTX. The protective effect of FNPS at 200 mg·(kg·d)^−1^ on IL-6 level was weaker than that at 100 mg·(kg·d)^−1^. This may be related to the inhibition of FNPS on tumor development.

### 3.6 Effects of FNPS on the phagocytosis and migration of RAW264.7 cells

**FIGURE 6:**
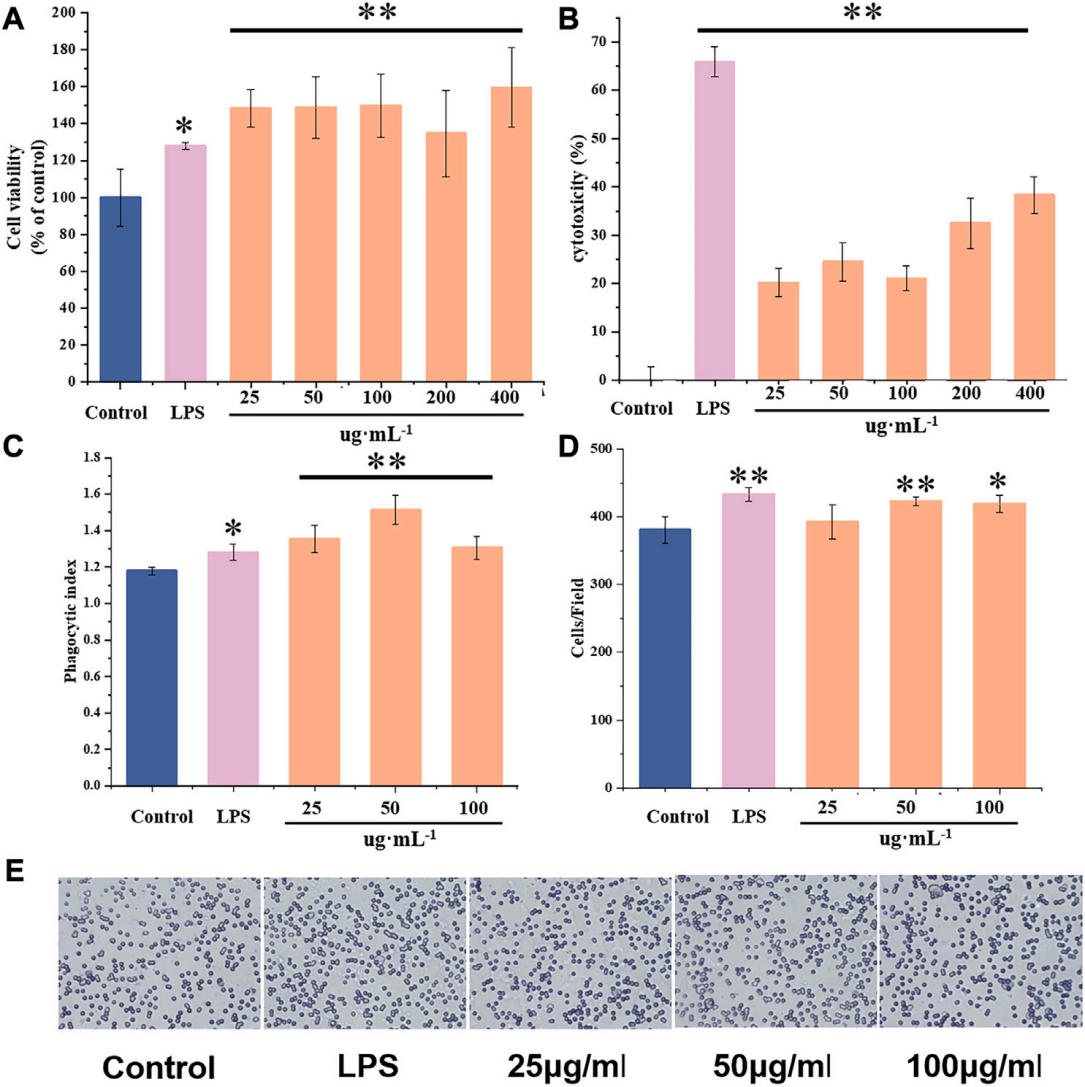
Effects of FNPS on the activity of RAW 264.7 cells **(A)** Effects of FNPS on the proliferation of RAW264.7 cells. **(B)** Cytotoxicity assay of FNPS in RAW264.7 cells. **(C)** Effects of FNPS on the phagocytosis of RAW264.7 cells. **(D)** Effects of FNPS on the migration of RAW264.7 cells, stained with crystal violet. **(E)** Representative images of RAW264.7 cells migration tests. Note: Control, unadministered cells; LPS, lipopolysaccharide *p < 0.05 *versus* Control, **p < 0.01 *versus* Control.

The CCK-8 method was used to determine the effect of FNPS on the proliferation of RAW264.7 cells (Figure 6A). The toxicity of FNPS to RAW 264.7 cells was detected by LDH release assay (Figure 6B). The results suggested that FNPS promoted cell proliferation in the range of 25–400 μg mL^−1^. The LDH release of cells was significantly changed after the treatment of FNPS higher than 200 μg mL^−1^. When the concentration reached 200 μg mL^−1^, the LDH level tended to be stable and had significant differences from other concentrations. Therefore, *in vitro* experiments were performed with FNPS lower than 200 μg mL^−1^.

To determine the effects of FNPS on the immune function of RAW264.7 cells, phagocytosis and migration were evaluated. Phagocytosis of macrophages is one of its main biological functions. Phagocytosis of foreign bodies and damaged cells is an important way for macrophages to exert immune function. The effect of FNPS on the phagocytosis of RAW264.7 cells was shown in Figure 6C. It was suggested that 25 μg mL^-1^ FNPS significantly enhanced the phagocytosis activity of RAW264.7 (*p* < 0.01), and activated immune regulation function. The principle of the Transwell experiment is that cells seeded in the upper chamber tend to migrate to the lower chamber with a high concentration of FBS. The effect of FNPS on the migration function of RAW264.7 cells was shown in Figures 6D, E. It was observed that 50 μg mL^-1^ FNPS significantly increased the number of cells in the lower chamber (*p* < 0.05). It could be seen that 25 μg mL^-1^ FNPS activated the phagocytosis activity of RAW264.7 cells. 50 μg mL^-1^ FNPS could promote the migration of RAW264.7 cells so that they could gather to participate in the immune response.

## 4 Discussion

A polysaccharide fragment (FNPS) from raw slices of Fuzi was isolated in this study. Molecular weight is 94 kDa, composed of rhamnose, arabinose, galactose, glucose, and mannose in a molar ratio of 0.008:0.017:0.018:0.908:0.048. Studies have been conducted to isolate polysaccharides with molecular weights of 10, 200, 400, and 6000 kDa from Fuzi and its products (Fu et al., 2022). Yang et al. (2020) isolated a kind of Fuzi polysaccharide with high molecular weight (6.29 × 106) from Fuzi decoction slices by hot water method. The immunomodulatory activity of Fuzi polysaccharide and its effect on gastrointestinal diseases caused by immunosuppression were studied, corresponding to the immunosuppressive model under normal condition. This study is more concerned with immunosuppressive side effects during disease treatment, corresponding to the immunosuppressive model in pathological states. In terms of immune regulation, FNPS showed protective effects on immune organs at a lower dose. In terms of structure, FNPS showed lower molecular weight and more complex monosaccharide composition. This suggests that smaller molecular weights may have better immunomodulatory activity.

Recent studies have demonstrated that polysaccharides can activate macrophages to produce immune-related molecules and can be utilized as a potential natural immune-enhancing agent (Yang et al., 2022). Gao et al. (2010) isolated a low molecular weight polysaccharide (13.3 kDa) from Fuzi by water extraction method. The study confirmed that Fuzi polysaccharide has anti-tumor activity and obvious immunomodulatory effect, and speculated that polysaccharide with higher molecular weight has better anti-tumor activity. Based on the antitumor and immunomodulatory activity of Fuzi polysaccharide, the effects of FNPS on chemotherapy mice were studied. FNPS could be used in combination with CTX in the chemotherapy of H22 tumor-bearing mice to enhance efficacy and reduce toxicity. The preliminary experiment results showed that 50 mg·(kg·d)^−1^ FNPS had no significant improvement in the immunosuppression induced by CTX. Therefore, 100 mg·(kg·d)^−1^ and 200 mg·(kg·d)^−1^ FNPS were selected for administration. The results also suggested that the dose of polysaccharides or herbal medicines had a significant effect on immune activity. In view of the effect of FNPS on chemotherapy mice, we further verified the immunomodulatory activity by macrophage functional test.

The mechanism of the combination of FNPS and CTX may be related to the activation of immune cells. The occurrence of inflammation is related to TNF-α, IL-1β, IL-6, and other cytokines. In the immunosuppressed state, the level of inflammatory factors will be significantly reduced. In cellular immunity, TNF-α and IL-1β are Th1-related immune factors. And in humoral immunity, IL-10 is a Th2-related immune factor (Shapouri-Moghaddam et al., 2018). In the chemotherapy of H22 tumor-bearing mice, it was found that Th1/Th2 balance in H22 tumor-bearing mice favored Th1 immunity, that is, exhibited an overall inflammatory response. After treatment with CTX, the balance favored Th2 immunity. After FNPS combined administration, the Th1/Th2 balance shifted toward Th1. Therefore, the resistance of FNPS to CTX immunosuppression may be related to the regulation of the Th1/Th2 balance. We will also further verify this inference.

It can be inferred from the above experimental results that the polysaccharide with lower molecular weight is more suitable for immunoregulatory activity, and the polysaccharide with higher molecular weight is more suitable for antitumor activity. Different polysaccharide fragments can be selected according to different needs to play a better curative effect. This study focused more on the protective effect of FNPS on immunosuppressive side effects induced by chemotherapy. The synergistic effect of FNPS on chemotherapy can be further studied. And it is a potential target for future research to modify the structure of polysaccharides extracted from Fuzi.

## 5 Conclusion

A neutral polysaccharide fragment from Fuzi was isolated. It was revealed that it has immunomodulatory activity and could promote an inflammatory response. The results showed that it could ameliorate the immunosuppressive side effects of chemotherapy in mice. The scientific basis of Fuzi for the treatment of liver cancer was partly explained. It provided a new perspective and evidence for improving the effectiveness and decreasing the toxicity of integrated traditional Chinese and Western medicine in tumor treatment. It is helpful to expand the application of Fuzi in the treatment of tumor diseases.

## Data Availability

The raw data supporting the conclusion of this article will be made available by the authors, without undue reservation.

## Abbreviations

CCK-8, cell counting kit-8; CTX, cyclophosphamide; SEM, scanning electron microscope; FBS, fetal bovine serum; FNPS, Fuzi neutral polysaccharide; FT-IR, Fourier transform infrared spectrometer; GPC, gel permeation chromatography; H22, Hepatoma 22; IC, ion chromatography; LDH, lactate dehydrogenase; LPS, lipopolysaccharide; PBS, phosphate buffer solution.
